# Evidence for Weakened Intercellular Coupling in the Mammalian Circadian Clock under Long Photoperiod

**DOI:** 10.1371/journal.pone.0168954

**Published:** 2016-12-22

**Authors:** M. Renate Buijink, Assaf Almog, Charlotte B. Wit, Ori Roethler, Anneke H. O. Olde Engberink, Johanna H. Meijer, Diego Garlaschelli, Jos H. T. Rohling, Stephan Michel

**Affiliations:** 1 Department of Molecular Cell Biology, Laboratory for Neurophysiology, Leiden University Medical Center, Leiden, The Netherlands; 2 Lorentz Institute for Theoretical Physics, Leiden University, Leiden, The Netherlands; University of Texas Southwestern Medical Center, UNITED STATES

## Abstract

For animals living in temperate latitudes, seasonal changes in day length are an important cue for adaptations of their physiology and behavior to the altered environmental conditions. The suprachiasmatic nucleus (SCN) is known as the central circadian clock in mammals, but may also play an important role in adaptations to different photoperiods. The SCN receives direct light input from the retina and is able to encode day-length by approximating the waveform of the electrical activity rhythm to the duration of daylight. Changing the overall waveform requires a reorganization of the neuronal network within the SCN with a change in the degree of synchrony between the neurons; however, the underlying mechanisms are yet unknown. In the present study we used PER2::LUC bioluminescence imaging in cultured SCN slices to characterize network dynamics on the single-cell level and we aimed to provide evidence for a role of modulations in coupling strength in the photoperiodic-induced phase dispersal. Exposure to long photoperiod (LP) induced a larger distribution of peak times of the single-cell PER2::LUC rhythms in the anterior SCN, compared to short photoperiod. Interestingly, the cycle-to-cycle variability in single-cell period of PER2::LUC rhythms is also higher in the anterior SCN in LP, and is positively correlated with peak time dispersal. Applying a new, impartial community detection method on the time series data of the PER2::LUC rhythm revealed two clusters of cells with a specific spatial distribution, which we define as dorsolateral and ventromedial SCN. Post hoc analysis of rhythm characteristics of these clusters showed larger cycle-to-cycle single-cell period variability in the dorsolateral compared to the ventromedial cluster in the anterior SCN. We conclude that a change in coupling strength within the SCN network is a plausible explanation to the observed changes in single-cell period variability, which can contribute to the photoperiod-induced phase distribution.

## Introduction

Anticipating seasonal changes in temperature and in food availability is important for the survival and reproductive success of many organisms. Seasonal changes are accompanied by latitude-dependent alterations in the duration of daylight, which in mammals is perceived by the eyes and subsequently transferred to several non-image-forming brain areas. One of these areas is the suprachiasmatic nucleus (SCN) located in the hypothalamus, directly above the optic chiasm. The SCN is considered to be the central mammalian pacemaker, responsible for circadian rhythmicity in physiology and behavior. The SCN receives direct projections from the retina, and light is found to be the most potent zeitgeber of circadian rhythmicity. Several studies indicate that the SCN is also involved in physiological adaptation to seasonal changes, like hibernation and breeding (reviewed in [1]). At the tissue level, the SCN responds to long summer days with a broadened peak in the waveforms of electrical activity [2] and gene expression rhythms [3]. It has become apparent that this decompression, observed at the ensemble level, is a consequence of a wider distribution of phases of single-cell oscillations after exposure to a long photoperiod [4, 5]. This increase in phase dispersion shows clear regional organization for gene expression over the rostrocaudal and dorsoventral axis [3, 4, 6–8].

A major question in the field of circadian rhythm research is how network-level phase synchrony and desynchrony are established. Phase desynchrony among SCN neurons can either be the result of poor coupling, or it can result from stable coupling in which phase differences are actively established. One example for the latter is observed in hamsters in constant light, who split their locomotor activity rhythms in two components that are usually 12 hours out of phase. It has been shown that the left and right SCN of these animals seem to be actively driven into a stable antiphase relation [9, 10]. In the case of aging, on the other hand, cells lose synchrony more likely as a result of a reduced coupling strength as evidenced by the weakening of coupling pathways like the reduction of GABAergic synaptic activity [11, 12] and a loss of VIP neurons in aged rodents [13, 14]. The question addressed in this study is if a change in coupling strength in the SCN circuitry could contribute to the adaptive physiological behavior in healthy young animals to long versus short day photoperiods [5]. If coupling is reduced by long photoperiod, this should be reflected in an increase in day-to-day variability in single-cell oscillatory patterns. If, on the other hand, phase desynchronization is achieved through an active mechanism, enhanced single-cell variability is not a prerequisite.

We used bioluminescence imaging of single-cell PER2::LUC gene expression rhythms to investigate whether the increased phase dispersal of electrical rhythms reported for long photoperiods is reflected in the phase distribution of single-cell PER2::LUC rhythms and if this is correlated to an increase in cycle-to-cycle period instability of individual neurons. We found that exposure to long days indeed led to a larger phase dispersion of PER2::LUC rhythms in the anterior SCN, which was associated with a significantly higher single-cell period variability. Further spatial analysis with an impartial method for clustering, which has previously been used to analyze different time series data [15, 16], revealed that the higher cycle-to-cycle period variability is restricted to a subset of cells located in the dorsolateral part of the anterior SCN. These results highlight the complex spatiotemporal organization of the SCN, in which specific regions of the SCN respond differently to photoperiod. Most importantly, the increased period variability in long photoperiod indicates that weakening of intercellular coupling contributes to the phase dispersal.

## Methods

### Animals and housing

The experiments in this study were performed in accordance to the Dutch law on animal welfare and approved by the Dutch government. Permit (DEC 11010) was issued by the animal ethical committee of the Leiden University Medical Center. Homozygous PERIOD2::LUCIFERASE (PER2::LUC) knock-in mice [17] were obtained from G. Lundkvist (Karolinska Institute, Stockholm, Sweden) and rederived at the animal facility of the Leiden University Medical Center using female wild type C57BL/6J (Charles River). This homozygous line was generated by backcrossing heterozygous knock-in mice to wild type C57BL/6J for more than 10 generations and then intercrossed. Male mice—average age 6.3 ± 0.6 months—were maintained in climate controlled cabinets equipped with full-spectrum diffused lighting of 50–100 lux intensity (Osram truelight TL). Mice had access to food and water ad-libitum, were kept in groups of 2–5 animals and had access to nesting material. Mice were subjected to either a long photoperiod (LP; 16 h light, 8 h dark) or a short photoperiod (SP; 8 h light; 16 h dark) schedule for at least 28 days. Animals were sacrificed within two hours before lights off, since dissection during that period is found to least affect the SCN rhythm [18, 19]. Experimental time was expressed as the projected external time (ExT), with ExT 0 representing the midnight, and ExT 12 the midday of the previous light regime.

### Bioluminescence imaging

Slice cultures of the SCN were prepared as described previously [20]. Briefly, mice were decapitated and the brain dissected and placed in ice cold, low Ca^2+^ and high Mg^2+^ artificial cerebrospinal fluid (ACSF), containing (in mM): NaCl (116.4), KCl (5.4), NaH_2_PO_4_ (1.0), MgSO_4_ (0.8), CaCl_2_ (1.0), MgCl_2_ (4.0), NaHCO_3_ (23.8), D-glucose (16.7) and 5 mg/l gentamicin (Sigma Aldrich) saturated with 95% O2−5% CO_2_ (pH 7.4). From each animal, two consecutive coronal slices (200 μm thick) of the hypothalamus containing the SCN were made with a VT 1000S vibrating microtome (Leica), the slice containing the anterior SCN was optically identified by using the third ventricle and the optic chiasm as landmarks (S1A Fig). The next slice was considered to contain the posterior SCN. The SCN was isolated and slice cultures were maintained on a Millicell membrane insert (PICMORG50, Millipore). In a subset of experiments the SCN tissue of two animals (four slices) were cultured on a single membrane insert. Membrane inserts were placed in a sealed 35 mm dish containing 1.2 ml of Dulbecco’s Modified Eagles Medium (D7777, Sigma-Aldrich) supplemented with 10 mM HEPES-buffer (Sigma-Aldrich), 2% B-27 (Gibco), 5 U/ml penicillin and 5 μg/ml streptomycin (0.1% penicillin-streptomycin, Sigma-Aldrich) and 0.2 mM D-luciferin sodium salt (Promega), adjusted to pH 7.2 with NaOH.

SCN slices were immediately transferred to an upright microscope (BX51WIF, Olympus) housed in a light-tight and temperature-controlled chamber, at 37°C (Life Imaging Services, Reinach, Switzerland). The microscope was equipped with a long-working distance objective (HN10X/22, Olympus), a cooled CCD camera (ORCA UU-BT-1024, Hamamatsu) and a motorized stage (XY-shifting table 240, Luigs & Neumann Ratingen, Germany) as well as focus control (MA-42Z, Märzhäuser, Wetzlar, Germany) both connected to an OASIS-4i Four Axis Controller. Bioluminescence images were obtained from either four or two SCN cultures using exposure times of 14.5 and 29 minutes respectively. Stage and focus position as well as image acquisition was controlled by Image Pro Plus software (MediaCybernetics, Warrendale PA USA; StagePro plug-in, Objective Imaging, Cambridge, UK).

### Luminescence data processing

A MATLAB-based (Mathworks, Natick, MA) custom-made program (source code available on request) was used to analyze the time series of images. First, cell-like regions of interest (ROIs) were defined by automated detection of groups of pixels with luminescence intensity above the noise level. The location of these cell-like ROIs had to be consistent throughout the recordings to include them for further analysis. For convenience we will refer to these cell-like ROIs as “single cells”. In order to reduce noise and increase the efficiency for subsequent analyses, a previously described algorithm [21] was used to smooth and resample the data to one data point per minute. This provides a sinus-like wave for each single cell. Finally, the smoothed data were detrended and normalized using a fourth order polynomial from the TSA toolbox for MATLAB.

The processed intensity traces from the single-cell rhythms were evaluated on sustained PER2::LUC signal and circadian rhythmicity. Single cells were only used if the time series contained at least three cycles, with peaks above and troughs below the trend line, and with an averaged interpeak interval in the circadian range of 20–28 hours. The selection procedure yielded on average 134 cells per slice, with a minimum of 56, and a maximum of 254 cells. Although the average number of cells in the anterior SCN was slightly higher than in the posterior SCN, there was no significant difference between any of the groups (S1E Fig).

To assess peak time and peak time variability, the first peak after the first trough of the PER2::LUC expression rhythm was used as a phase marker. Peak time variability was defined as the standard deviation (SD) of the peak times from all cells per slice.

We determined the cycle-to-cycle interval between the half-maximum of the rising edge of the PER2::LUC expression rhythm and calculated the day-to-day variability in period in accordance to previous studies using this parameter as an indication for period stability and precision [22, 23]. We defined the period variability as the standard deviation (SD) of the cycle-to-cycle interval of individual cells [23]. We calculated the period variability for the first three cycles for each cell in a slice, and averaged the single-cell period variability of all cells per slice.

Data were further analyzed using GraphPad Prism software. For comparison between groups, a two-way ANOVA was used, followed by a Sidak post hoc analysis for significance. The correlation between peak time SD and period SD was analyzed with a linear regression.

### Spatial pattern distribution

A spatial analysis of the variability in period of single cells in the SCN was performed. From a binary representation matrix of both the left and right SCN, the center of mass—which is the weighted average position of mass in a body or a system of particles—was calculated using the following mathematical formula:

$$x_{cm}=\sum x_{i}m_{i}/M\;;\;\;y_{cm}=\sum y_{i}m_{i}/M$$

where x_cm_, y_cm_ is the position of centre of mass, x_i_, y_i_ is the position of the i-th particle with mass m_i_ and M is the total mass of the body. Based on these centers, each SCN was divided into six regions and all ROIs within each region analyzed. For each slice, the average single-cell period SD was calculated per region and weighted for the corresponding number of RIOs. We then calculated the weighted average for all slices of each experimental group (LP anterior and posterior, SP anterior and posterior). This resulted in average regional values for period variability for every experimental group for six regions in both the left and the right SCN.

### Community detection

To resolve functional modules in the SCN neuronal network, a recently developed advanced method for the identification of community structure was employed [15, 24]. First, a cross-correlation matrix from the multiple time series of the bioluminescence intensity traces was constructed. Next, using random matrix theory, both the local (neuron-specific) noise and global (SCN-wide) dependencies from the correlation matrix were filtered out [15]. In contrast to alternative community detecting methods, there is no need to introduce arbitrary thresholds. The method leads to the identification of functional modules (communities) that are guaranteed to have positive overall correlation within communities and negative overall correlation between communities, relative to the global SCN activity.

## Results

Single-cell PER2::LUC expression rhythms were measured in slice cultures from PER2::LUC mice entrained to LP or SP (S1A Fig). In the first set of imaging recordings, using an exposure time of 14.5 minutes, we observed a lower amplitude of PER2::LUC rhythms in the anterior SCN in LP compared to SP (S1 Fig; all data obtained with this exposure time are shown in the Supplemental Figures). To ensure that a low signal-to-noise ratio would not compromise the data analysis of the peak time distribution, we performed a second set of experiments with a longer exposure time (29 minutes). This resulted in higher amplitude PER2::LUC rhythms (S1D Fig), but all principal findings on peak time distribution and period variability were consistent between the two data sets (S2 Fig).

### Phase relationship between cells of the anterior and posterior SCN

**Fig 1:**
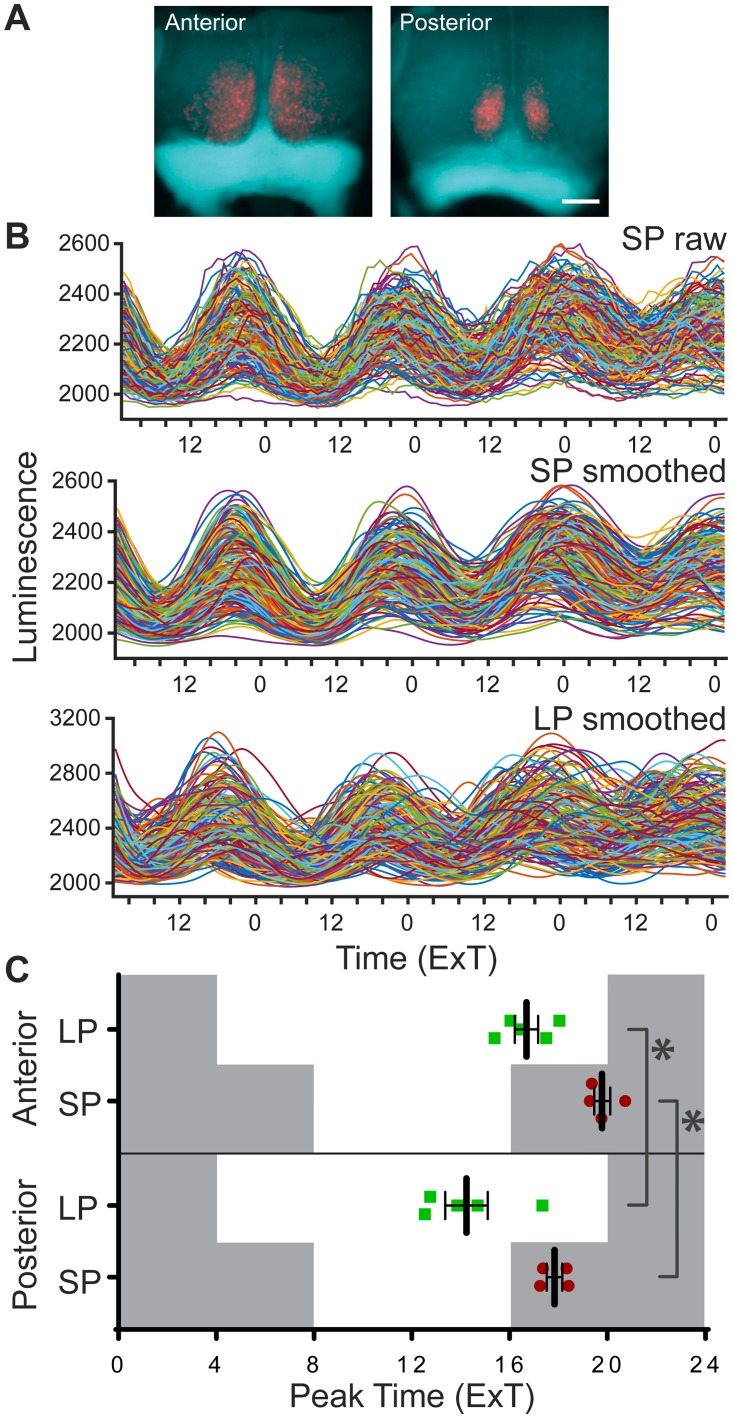
Peak time of PER2::LUC expression is similar in anterior and posterior SCN in both long and short photoperiod. (A) Overlays of brightfield images of anterior SCN cultures from short (SP) and long photoperiod (LP), with the corresponding bioluminescence image. Scale bar: 200 μm. (B) Intensity traces of PER2::LUC expression from single cells. Raw traces of bioluminescence intensity from the anterior SCN from SP (*n* = 183 cells; top panel), with corresponding smoothed traces (middle panel), and smoothed traces from the anterior SCN from LP (*n* = 177 cells; bottom panel). (C) Average peak time of PER2::LUC rhythms per slice, of the anterior and posterior SCN in LP (green squares, *n* = 5) and SP (red circles, *n* = 4) are plotted as external time (ExT). Grey background indicates projected dark period of light regime preceding the experiment. Black bars indicate mean ± SEM; * *p* < 0.05.

To assess the phase relationship between the anterior and posterior SCN in both LP and SP conditions, PER2::LUC peak time was determined from smoothed bioluminescence intensity traces of the single cells, and averaged per slice (Fig 1A and 1B). The average peak time–expressed in ExT–was about two hours later in the anterior, compared to the posterior slice, for both LP and SP (Fig 1C; LP, anterior: ExT 16.7 ± 0.5, *n* = 5, posterior: ExT 14.2 ± 0.9, *n* = 5; *p* < 0.05; SP, anterior: ExT 19.8 ± 0.3, *n* = 4, posterior ExT 17.8 ± 0.3, *n* = 4; *p* < 0.05; cf. S2A Fig). In LP, all average peak times per slice preceded the projected time of lights-off, while in SP all average peak times followed after the projected time of lights-off (Fig 1C). The data on peak time show that the temporal relationship of anterior to posterior SCN is maintained in different photoperiods.

### Peak time dispersion among cells along the anterior-posterior axis

**Fig 2:**
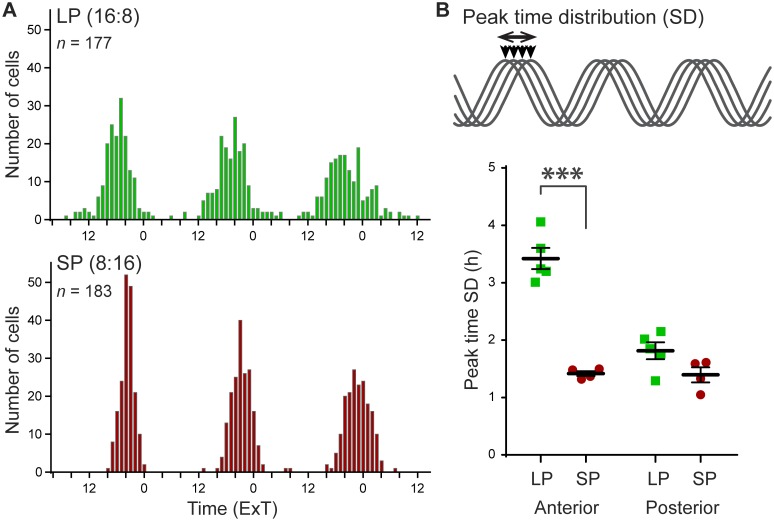
Exposure to long photoperiod increases peak time distribution. (A) Representative histograms of peak times of two individual slices from the anterior SCN in long (LP, *n* = 177 cells) and short photoperiod (SP, *n* = 183 cells) plotted in external time (ExT). (B) Phase distribution is defined as the standard deviation (SD) of peak time, of the first cycle *in vitro* (top panel). Phase distribution was calculated per slice, and is shown for the anterior and posterior SCN (bottom panel), in LP (green squares) and SP (red circles). Black bars indicate mean ± SEM; *** *p* < 0.001.

To test whether photoperiod affects the phase relationship among cells, we used the standard deviation (SD) of peak times as a measure of phase dispersion within slices (Fig 2B). Photoperiod had a clear effect on the distribution of peak times in cellular PER2::LUC rhythms (Fig 2A). The SD of peak times was significantly higher in LP than in SP, but only in the anterior SCN (Fig 2B; anterior, LP: 3.42 ± 0.19, *n* = 5, SP 1.42 ± 0.04, *n* = 4; *p* < 0.001; posterior, LP: 1.81 ± 0.15, *n* = 5, SP: 1.40 ± 0.13, *n* = 4; *p* > 0.05; cf. S2B Fig). This photoperiod-induced difference in phase dispersion was not dependent on the time in culture (S3 Fig). The results emphasize the spatial heterogeneity in photoperiodic response within the SCN.

### Single-cell period variability differences along the anterior-posterior axis

**Fig 3:**
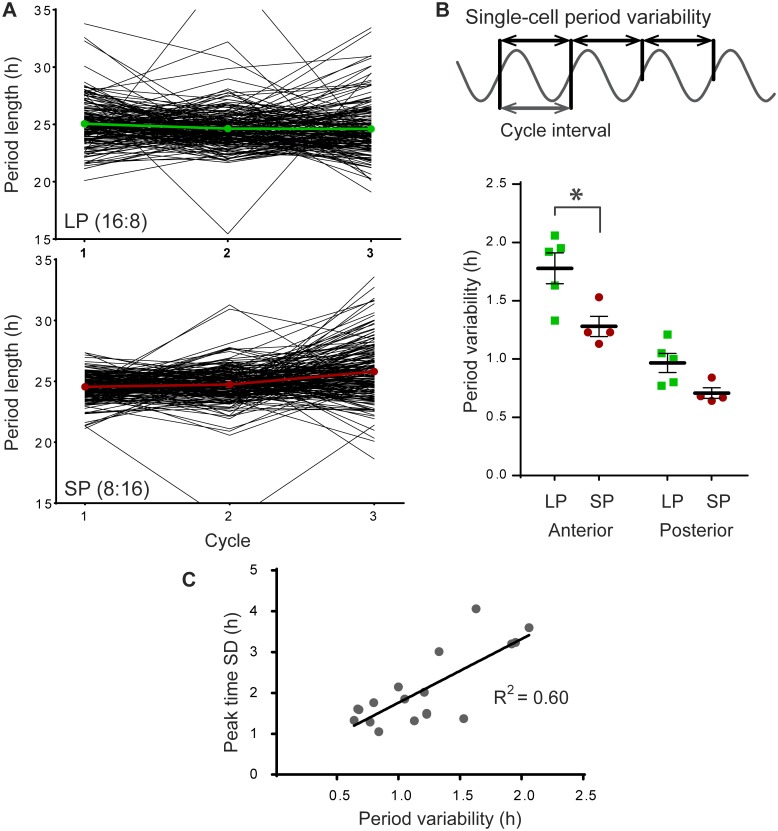
Single-cell period variability (SD) increases after exposure to long photoperiod. (A) Representative examples of the variability in single-cell cycle-to-cycle interval from anterior SCN slices in long (LP, *n* = 177; top panel) and in short photoperiod (SP, *n* = 183; bottom panel). Black traces show the cycle-to-cycle interval of individual cells for the first three cycles *in vitro*; colored traces show the average period per cycle for the presented slice. (B) Cycle interval is defined as the cycle-to-cycle time difference between the half-maximum of the rising edge of the PER2::LUC expression rhythm. The variability in period is defined as the standard deviation (SD) of the cycle interval of individual cells, calculated for the first three cycles *in vitro* (top panel). Single-cell period variability was averaged per slice and is shown for the anterior SCN, in LP (green squares) and SP (red circles; bottom panel). Black bars indicate mean ± SEM. (C) Linear regression of the relationship between single-cell period variability and peak time SD for all recordings (*n* = 18, *p* < 0.001).

We next sought to determine whether an increase in period variability of single cells could contribute to an increase in peak time dispersion. The fluctuation of the cycle-to-cycle period of individual cells was analyzed (Fig 3A). The standard deviation (SD) of the cycle intervals of the first three cycles of PER2::LUC rhythms was determined for individual cells, and averaged per slice (Fig 3B). The average variability in single-cell period was increased in LP compared to SP in the anterior SCN (*p* < 0.05), and in anterior vs. posterior in both long and short photoperiod (*p* < 0.001 and *p* < 0.01 respectively; anterior, LP: 1.78 ± 0.30, *n* = 5, SP: 1.28 ± 0.17, *n* = 4; posterior, LP: 0.97 ± 0.18, *n* = 5, SP: 0.71 ± 0.10, *n* = 4; Fig 3B), meaning that the cycle interval of individual cells varied on average 0.5 hours per cycle more in LP than in SP. It should be noted that the average period of the cellular PER2::LUC rhythm over 3 days was not affected by photoperiod (S4B Fig). Increasing period variability of single cells could be a mechanism to achieve peak phase dispersion. Indeed, assessment of the relation between phase dispersion and single-cell period variability showed that peak-time SD and the single-cell period SD was strongly correlated (*R*^*2*^ = 0.60, *p* < 0.001; Fig 3C; cf. S2D Fig).

### Regional differences in single-cell period variability in the coronal plane

**Fig 4:**
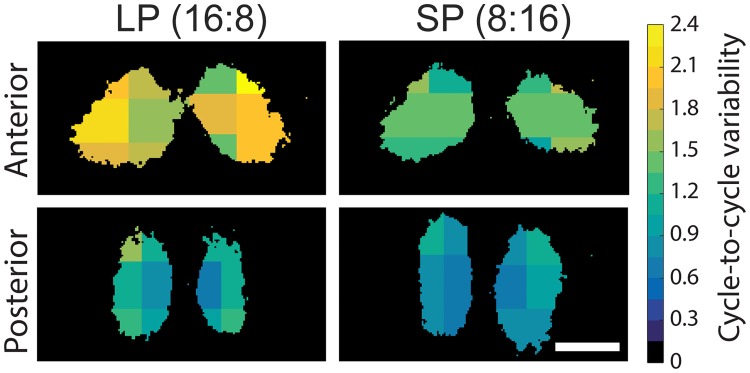
Single-cell period variability (SD) shows regional differences, especially after exposure to long photoperiod. Regional averages of single-cell period variability binned from all recordings for six areas of the anterior (top panels) and posterior SCN (bottom panels), in long (LP, left panels) and short photoperiod (SP, right panels). As indicated by the color bar on the right, dark (blue-black) colors indicate small, and light (green-yellow) colors indicate larger period variability. Scale bar: 200 μm.

To map the spatial heterogeneity of period variability within slices, we arbitrarily divided the SCN in six regions for which the single-cell period variability was averaged (Fig 4). We found only minor differences between the regions in period variability in SP, which could be expected from the overall lower variability in phases in this photoperiod. Interestingly, the differences between the regions were more substantial in LP. The average variability of single cells was found to be lower in the medial than in the lateral region of the SCN. Furthermore, the differences were consistently larger in the anterior SCN compared to the posterior SCN.

### Characterization of functional clusters of neurons within the SCN

**Fig 5:**
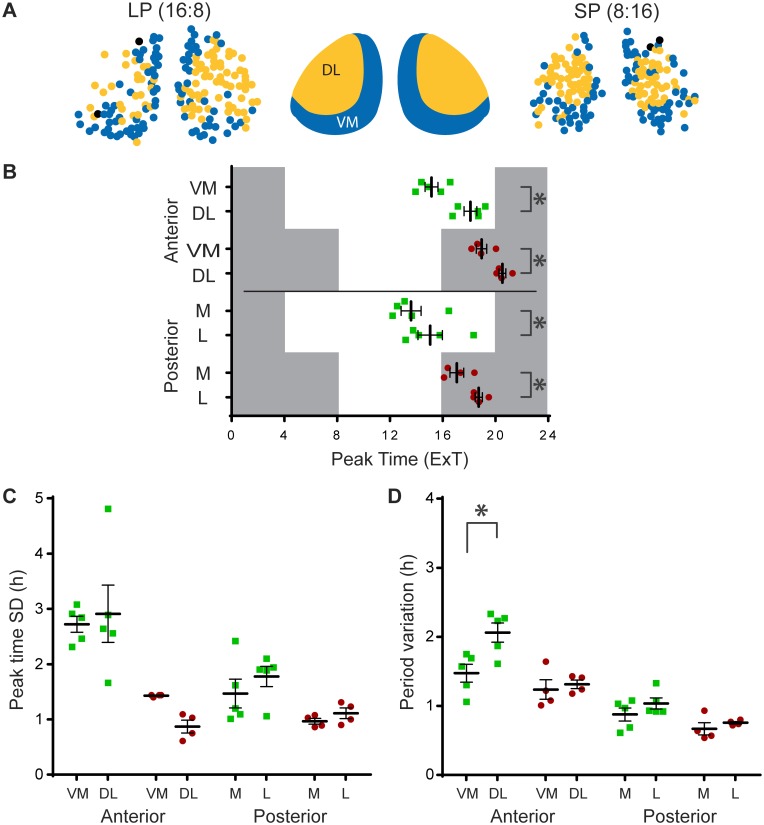
Functional clusters show distinct spatial distribution and region specific rhythm characteristics. (A) Representative examples of the communities detected by an advanced, unsupervised method for correlation matrix analysis [15]. The community detection method automatically divided the SCN in two distinct regions, a ventromedial (VM) and dorsolateral (DL) region in the anterior SCN (middle panel), and a medial (M) and lateral (L) region in the posterior SCN, in both long (LP; left panel), and short photoperiod (SP, right panel). (B) Peak times in external time (ExT) were averaged per region, per slice. Darkness and light of the previous light regime are represented by grey and white background respectively. (C) Phase distribution, defined as peak time standard deviation (SD), was calculated per region, per slice. (D) Single-cell period variability was averaged per region, per slice. All data are shown for the VM and DL region in the anterior, and M and L region in the posterior SCN, in LP (green squares) and SP (red circles). Black bars indicate mean ± SEM; * *p* < 0.01.

To extend the analysis of regional differences in the coronal plane and identify clusters of cells expressing similar dynamic behavior, we employed a recently developed community detection approach designed specifically for correlation matrices [15, 16]. This method consistently yielded two main groups of cells, which displayed a robust spatial distribution within both the anterior and posterior slice. This correlation-based differentiation yielded a similar spatial distribution as described above. More specifically, in the anterior slice the communities divided the SCN in a ventromedial (VM) region and a dorsolateral (DL) region (Fig 5A). Note that these regions do not correspond with the division of the SCN in dorsomedial (shell) and ventrolateral (core) SCN defined originally in rats by the distribution of neuropeptide content [25], but later revised for mice [26]. In the posterior slice the two communities were found in the medial (M) and lateral (L) regions of the SCN. We characterized these clusters in peak time, phase dispersion and single-cell period variability. The VM in the anterior, and the M region in the posterior SCN peaked earlier compared to the DL and L region (Fig 5B; anterior LP: DL: ExT 18.1 ± 0.5; VM: ExT 15.2 ± 0.5, *n* = 5, *p* < 0.01; SP: DL: ExT 20.5 ± 0.3; VM: ExT 19.0 ± 0.4, *n* = 4, *p* < 0.01; posterior LP: L: ExT 15.1 ± 0.9; M: ExT 13.6 ± 0.8, *n* = 5, *p* < 0.01; SP: L: ExT 18.8 ± 0.3 M: ExT 17.1 ± 0.5, *n* = 4, *p* < 0.01). With respect to phase dispersion, there were no differences between the regions (Fig 5C). Interestingly, in the anterior SCN in LP, the single-cell period variability was higher in the DL region, compared to the VM region (Fig 5D; LP, anterior, DL: SD 2.06 ± 0.31, VM: SD 1.47 ± 0.29, *n* = 5, *p* < 0.001). The spatial distribution of the two groups of cells identified with the impartial method of community detection is similar to the pattern defined by physiological parameters (cf. Fig 4).

## Discussion

Plasticity of the neuronal network within the SCN is necessary for day-length encoding and proper re-entrainment, but the underlying detailed mechanisms are still largely elusive [27]. We analyzed PER2::LUC gene expression rhythms of single SCN neurons of mice exposed to different photoperiods to investigate the dynamic behavior of clock neurons at different synchronized states. Exposure to long photoperiod (LP) resulted in an increase in peak time distribution of single-cell PER2::LUC rhythms in the anterior part of the SCN, compared to short photoperiod (SP). Interestingly, the peak time distribution was positively correlated with the jitter in PER2::LUC period. Spatial analysis of the period variability revealed that the largest variability in cycle-to-cycle period were located in the dorsolateral part of the SCN. Moreover, an impartial community detection approach revealed a similar spatial distribution of neuronal intrinsic oscillator features in different photoperiods. The data suggest that photoperiod-induced changes in intrinsic features of cellular rhythmicity, like the stability of the period, contribute to the phase distribution required for encoding day length in a distinct subpopulation of the SCN. These findings support the view that phase desynchrony is a result of the reduction in coupling between single-cell oscillators allowing for a subsequent emergence of cellular divergences.

### Population peak time and peak phase

In the present study we observed a difference in mean peak time of PER2::LUC rhythms between the anterior and posterior SCN, with the posterior SCN peaking earlier than the anterior SCN (Fig 1C). This is consistent with previous studies on gene expression rhythms in the anterior vs. posterior SCN [3, 7, 28], however see [29].

Exposure to LP, but not to SP, led to considerably larger phase differences in PER2::LUC expression rhythms between the single cells of the anterior, compared to posterior SCN (Figs 1B and 2), which is in accordance with previous studies on single-cell recordings in *Per1* expression [4]. In addition, both PER2::LUC and Bmal1-ELuc recordings from mice exposed to LP also revealed an increased regional phase dispersion in the (anterior) SCN, suggesting a broader influence of environmental photoperiod on the transcription-translation feedback loop [6, 30]. To our knowledge, this is the first study showing that the photoperiod-induced PER2 peak time distribution of single cells is restricted to the anterior SCN (Fig 2B). Notably, in the anterior SCN the difference in average peak time standard deviation between LP and SP is about two hours, suggesting it may strongly contribute to the photoperiod-induced broadening of the electrical activity rhythm in the SCN [5].

### Single-cell period variability

The increase in peak phase dispersion in LP can be established in three ways: (1) by increased phase dispersion between cells achieving a new stable phase relationship, but maintaining the same period; (2) by variability in period between individual cells, but with little instability in the period of the single cell; or (3) by variability in the cycle-to-cycle period of single cells (see insets in Figs 2B, 3B and S5). We hypothesize that coupling within the network is a potential mechanism that will both affect the accuracy of the period in single cells (option 3) as well as the variability of the period between the cells (option 2). Although there is increasing evidence for a stable phase relationship of gene expression rhythms between regions in the SCN (option 2) in different photoperiods [6, 28, 30, 31], we were interested to know if single-cell period variability (option 3) may contribute to peak phase dispersion. We therefore analyzed the period standard deviation, and found that it was increased in LP in the anterior SCN. Interestingly, the variability in period of the first three cycles in vitro was positively correlated with the peak time distribution in the first cycle (Fig 3C). Thus, a larger peak time distribution in the first cycle seems to be predictive of a large variability in cycle-to-cycle period. The increased single-cell period variability from cycle to cycle may indicate weak coupling among the cells. In this context it should be noted that period stability and the resulting high precision of the overt rhythms are a hallmark of the circadian clock [22, 32]. However, this seems to be primarily a result of a coupled network consisting of less accurate single cell clocks [23]. We therefore propose that photoperiod-induced variability in cycle-to-cycle period of the cells in the network is a consequence of a change in coupling strength, which contributes to the alteration in phase dispersal.

### Spatial heterogeneity in cellular rhythm properties

We next sought to determine whether the cycle-to-cycle period variability was spatially distributed in the SCN. Indeed, the mapping of regional averages of period variability revealed that the largest variability was found in the lateral part of the SCN (Fig 4). Notably, this was the case throughout the rostrocaudal plane, with the most pronounced differences in LP, in the anterior SCN. This spatial distribution coincides with the phase distribution found by Evans and colleagues [28], who reported a ~1.5 hour phase difference between the ventrolateral and dorsomedial part of the anterior SCN.

Subsequently we used an impartial community detection approach, which could identify functional clusters of cells, without knowledge on spatial locations [15]. Surprisingly, this method persistently identified two populations of neurons, of which the locations coincided largely with the regions described above (Fig 5A). While the cluster of cells in the ventromedial (VM) region of the anterior SCN, as well as the clusters located in the posterior SCN are more rigid in their oscillatory behavior, the clusters of cells found in the dorsolateral (DL) region of the anterior SCN show more oscillatory variability, raising the possibility that neurons in the DL and VM region of the anterior SCN are differentially affected by long photoperiod. The location of clusters defined by our community detection method overlap only partially with previously defined regions of the SCN based on neuropeptide content. Nevertheless, they are comparable to the regions described by Abel and colleagues [33], who found hubs in a small-world network located in the central SCN, corresponding to our DL cluster, and only sparsely connected cells in a region similar our VM cluster. Together with the results from the current study, this could mean that the DL network functions differently than the VL network, and responds differently to photoperiodic input.

Several previous studies have used clustering methods to identify functional groups of neurons within the SCN. Most methods require the specification of an arbitrary threshold value for inter-neuron correlation in order to project correlation matrices to networks and subsequently identify clusters within these networks. The resulting clusters are therefore unavoidably threshold-dependent. As an additional limitation, such thresholding methods tend to detect only the single cluster with the largest internal correlation, thereby failing to identify additional structures. The advantage of the method used in the current study is that no definition of an arbitrary threshold is needed. Two previous papers described clustering of “super-pixels” from imaging of PER2::LUC in the SCN, in which a *k-medoid* cluster analysis with the gap statistic with a cosine distance function was used to produce a pre-defined number (5) of clusters [34, 35]. Since these studies use super-pixels and we use cell-like ROIs it is difficult to directly compare the results, although the locations of the clusters that were found did not contradict the spatial organization of our clusters. Myung and co-workers [31] also used cluster analysis to identify regions with different temporal relationships; the spatial distribution of clusters found using two independent methods was only similar to the present study in the anterior SCN. The discrepancies may result from differences in rhythm dynamics between PER2 and BMAL1 expression as already suggested by the authors [31].

### Effect of photoperiod on coupling

This study shows for the first time that exposure to long photoperiod affects rhythm stability of single cells. This effect of photoperiod on single-cell period variability can be caused by different mechanisms and on different levels. On the single-cell level, period precision can be reduced by increased molecular noise [36]. At the network level, changes in the intercellular connectivity could result in period variability [23, 37]; changes in intercellular signaling could actively induce variability in the period of single cells [30]. Alternatively, reduced connectivity, meaning reduced intercellular signaling could cause larger “drifts” in period, before the signal is strong enough to correct a drifting cell.

Studies using computational modeling to evaluate the effect of molecular noise on circadian rhythmicity aimed at explaining the robustness of circadian rhythms in the SCN. This work shows that it takes considerably low gene expression of the core clock genes to affect period variability [36, 38, 39]. Although we cannot exclude a role of molecular noise in the period variability found in this study, our results do not suggest an effect of photoperiod on the amplitude in single-cell gene expression of PER2::LUC as would be predicted by the models (S1B and S1D Fig).

Previous experimental and modeling work suggests that alterations in the balance between attractive and repulsive coupling can induce a phase gap in BMAL expression rhythms between the dorsal and ventral region of the SCN [30, 40]. While the underlying neuronal mechanisms of photoperiod-induced phase adjustments within the SCN network is still not fully understood, there is mounting evidence for a role of GABA in dorsal-ventral communication, and seasonal encoding in the SCN [6, 30, 41]. However, the increased phase dispersion we found in the current study is present within, not between those regions, making it conceivable that other mechanisms are at play. The model of Myung and colleagues [30] is able to explain the emergence of a phase gap between different regions in the SCN, based on differences in period. However, it does not explain the period variability of single cells we see in this study. For that, a daily variability in attractive/repulsive coupling should take place between single SCN neurons and not only regions, which would be mechanistically challenging to explain.

Several studies have shown that reduced intercellular contact results in increased single-cell period variability [23, 37]. These studies have found that low-density plating of SCN neurons results in higher period variability in both electrical activity [37] and in Per1 gene expression [23], compared to cultured slices or high-density plating, respectively. These studies indicate that the increased period variability found after exposure to long photoperiod could result from reduced intercellular connectivity. These findings make it likely that the increased single-cell period variability documented in the present study is the result of reduced coupling within the SCN neuronal network.

## Conclusion

The results from this study suggest weakened intercellular coupling within the SCN after exposure to long photoperiods. Exposure to LP induced peak time dispersal in the anterior SCN, which was accompanied by single-cell period variability. Analysis of overall rhythm characteristics revealed that in LP the dorsolateral region of the SCN showed the largest period variability. We propose that exposure to long days locally reduces intracellular coupling and contributes to the mechanism controlling phase distribution in different photoperiods.

## Supporting Information

S1 Fig Longer exposure time increases signal-to-noise ratio of PER2::LUC rhythms. (A) Brightfield image of SCN slice culture. Dotted lines indicate third ventricle (3V) and optic chiasm (OC). (B) PER2::LUC Intensity traces from single-cells imaged with 14.5 min exposure time (cf. Fig 1B for 29 min exposure time). Raw bioluminescence intensity traces and corresponding smoothed traces from short photoperiod (SP; anterior; *n* = 131 cells; top and middle panel), and smoothed traces from long photoperiod (LP; anterior; *n* = 65 cells; bottom panel). (C) Average amplitude and (D) number of cells from recordings with 14.5 min exposure time, for both LP (green squares, anterior & posterior: n = 6), and SP (red dots; anterior: n = 5; posterior: n = 8). (E) Amplitude and (F) number of cells from the recordings with 29 min exposure time, for LP (green squares; n = 6), and SP (red dots; n = 4). Black bars indicate mean ± SEM.
(PDF)

S2 Fig Effect of photoperiod on PER2::LUC rhythm recorded with 14.5 min exposure time. (A) Average peak time of PER2::LUC rhythms are shown for the anterior and posterior SCN in LP (green squares) and SP (red circles). Peak time is plotted as external time (ExT). Dark period of the previous light regime are represented by grey background (cf Fig 1C). (B) Peak time distribution is defined as the standard deviation (SD) of the peak times per slice (cf. Fig 2B). (C) Period variability is defined as the SD of the period first three cycles in vitro per cell, and averaged per slice (cf. Fig 3B). (D) Correlation of the peak time SD and Period SD from all data of this set (cf. Fig 3C). Black bars indicate mean ± SEM.
(PDF)

S3 Fig Time in culture does not affect photoperiodic induced difference in phase dispersal. Peak time distribution (SD) was calculated as a measure for phase dispersal for the first three days in culture. Photoperiod effect on phase dispersal did not alter during the time in culture.
(PDF)

S4 Fig Period of the PER2::LUC expression rhythms. Period was determined over three cycles and averaged per slice, for (A) the experiments with 14.5 minutes exposure time, (B) 29 minutes exposure time. Black bars indicate mean ± SEM.
(PDF)

S5 Fig Cycle-to-cycle jitter in cells is larger in long photoperiod. Examples of PER2::LUC bioluminescence of six cells in LP (left) and SP (right) are shown, where the detrended signal of each individual cell (red) is compared to a sinusoid wave with the average period of the same cell (blue). The detrended signal is obtained by first smoothing the raw data. Then a fourth-order polynomial trend line was derived from the smoothed signal and subsequently subtracted. The sinusoidal signal was aligned to the half-maximum value of the rising edge of the first cycle. The time difference of the half-maximum values of the rising edge of subsequent cycles between the detrended data and the sinusoid represents the jitter in cycle-to-cycle interval. For each example, the number of the cell is shown in the upper right corner. Time is specified in ExT.
(PDF)
