# Supplementary material for: Evidence for Weakened Intercellular Coupling in the Mammalian Circadian Clock under Long Photoperiod
Source: PLoS One. 2016 Dec 22;11(12):e0168954. doi: 10.1371/journal.pone.0168954 (PMC5179103; doi:10.1371/journal.pone.0168954)
Supplement: S1 Fig — (A) Brightfield image of SCN slice culture. Dotted lines indicate third ventricle (3V) and optic chiasm (OC). (B) PER2::LUC Intensity traces from single-cells imaged with 14.5 min exposure time (cf. Fig 1B for 29 min exposure time). Raw bioluminescence intensity traces and corresponding smoothed traces from short photoperiod (SP; anterior; n = 131 cells; top and middle panel), and smoothed traces from long photoperiod (LP; anterior; n = 65 cells; bottom panel). (C) Average amplitude and (D) number of cells from recordings with 14.5 min exposure time, for both LP (green squares, anterior & posterior: n = 6), and SP (red dots; anterior: n = 5; posterior: n = 8). (E) Amplitude and (F) number of cells from the recordings with 29 min exposure time, for LP (green squares; n = 6), and SP (red dots; n = 4). Black bars indicate mean ± SEM. (PDF) [file pone.0168954.s001.pdf]

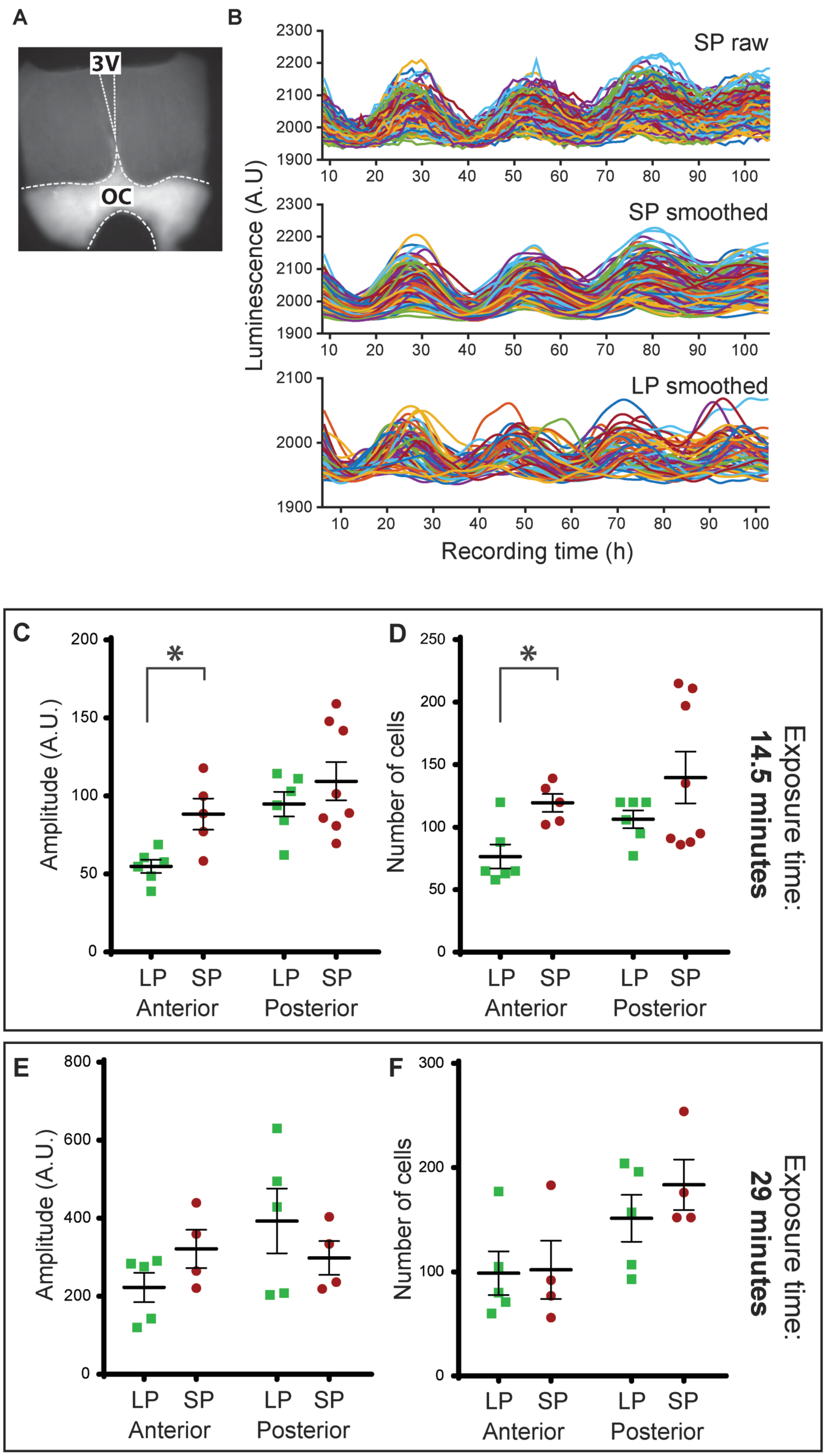

S1 Fig. Longer exposure time increases signal-to-noise ratio of PER2::LUC rhythms. (A) Brightfield image of SCN slice culture. Dotted lines indicate third ventricle (3V) and optic chiasm (OC). (B) PER2::LUC Intensity traces from single-cells imaged with 14.5 min exposure time (cf. Fig 1B for 29 min exposure time). Raw bioluminescence intensity traces and corresponding smoothed traces from short photoperiod (SP; anterior; n = 131 cells; top and middle panel), and smoothed traces from long photoperiod (LP; anterior; n = 65 cells; bottom panel). (C) Average amplitude and (D) number of cells from recordings with 14.5 min exposure time, for both LP (green squares, anterior & posterior: n = 6), and SP (red dots; anterior: n = 5; posterior: n = 8). (E) Amplitude and (F) number of cells from the recordings with 29 min exposure time, for LP (green squares; n = 6), and SP (red dots; n = 4). Black bars indicate mean ± SEM.
