# Supplementary material for: Evidence for Weakened Intercellular Coupling in the Mammalian Circadian Clock under Long Photoperiod
Source: PLoS One. 2016 Dec 22;11(12):e0168954. doi: 10.1371/journal.pone.0168954 (PMC5179103; doi:10.1371/journal.pone.0168954)
Supplement: S2 Fig — (A) Average peak time of PER2::LUC rhythms are shown for the anterior and posterior SCN in LP (green squares) and SP (red circles). Peak time is plotted as external time (ExT). Dark period of the previous light regime are represented by grey background (cf Fig 1C). (B) Peak time distribution is defined as the standard deviation (SD) of the peak times per slice (cf. Fig 2B). (C) Period variability is defined as the SD of the period first three cycles in vitro per cell, and averaged per slice (cf. Fig 3B). (D) Correlation of the peak time SD and Period SD from all data of this set (cf. Fig 3C). Black bars indicate mean ± SEM. (PDF) [file pone.0168954.s002.pdf]

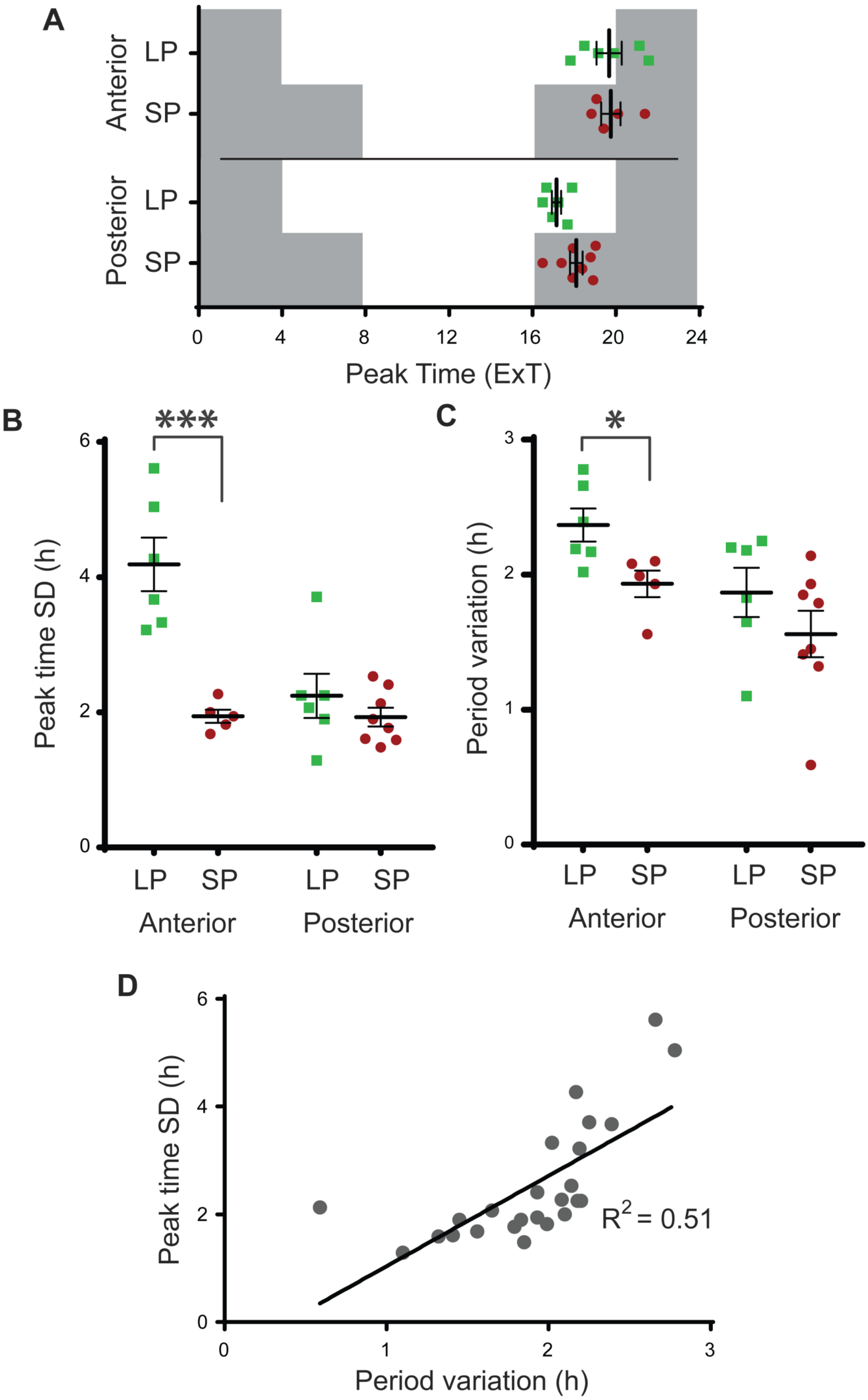

S2 Fig. Effect of photoperiod on PER2::LUC rhythm recorded with 14.5 min exposure time. (A) Average peak time of PER2::LUC rhythms are shown for the anterior and posterior SCN in LP (green squares) and SP (red circles). Peak time is plotted as external time (ExT). Dark period of the previous light regime are represented by grey background (cf Fig 1C). (B) Peak time distribution is defined as the standard deviation (SD) of the peak times per slice (cf. Fig. 2B). (C) Period variability is defined as the SD of the period first three cycles in vitro per cell, and averaged per slice (cf. Fig 3B). (D) Correlation of the peak time SD and Period SD from all data of this set (cf. Fig 3C). Black bars indicate mean ± SEM.
