# Supplementary material for: Evidence for Weakened Intercellular Coupling in the Mammalian Circadian Clock under Long Photoperiod
Source: PLoS One. 2016 Dec 22;11(12):e0168954. doi: 10.1371/journal.pone.0168954 (PMC5179103; doi:10.1371/journal.pone.0168954)
Supplement: S3 Fig — Peak time distribution (SD) was calculated as a measure for phase dispersal for the first three days in culture. Photoperiod effect on phase dispersal did not alter during the time in culture. (PDF) [file pone.0168954.s003.pdf]

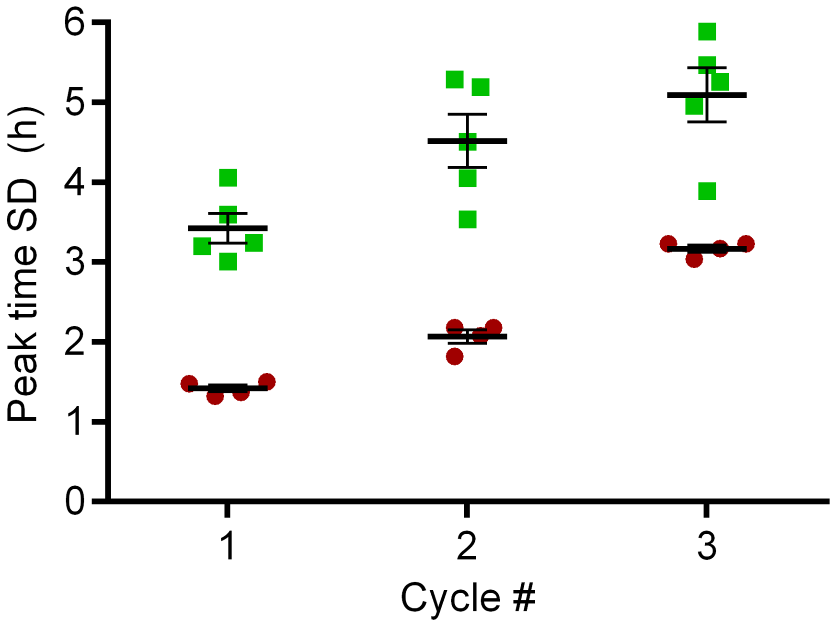

S3 Fig. Time in culture does not affect photoperiodic induced difference in phase dispersal. Peak time distribution (SD) was calculated as a measure for phase dispersal for the first three days in culture. Photoperiod effect on phase dispersal did not alter during the time in culture.
