# Supplementary material for: Evidence for Weakened Intercellular Coupling in the Mammalian Circadian Clock under Long Photoperiod
Source: PLoS One. 2016 Dec 22;11(12):e0168954. doi: 10.1371/journal.pone.0168954 (PMC5179103; doi:10.1371/journal.pone.0168954)
Supplement: S4 Fig — Period was determined over three cycles and averaged per slice, for (A) the experiments with 14.5 minutes exposure time, (B) 29 minutes exposure time. Black bars indicate mean ± SEM. (PDF) [file pone.0168954.s004.pdf]

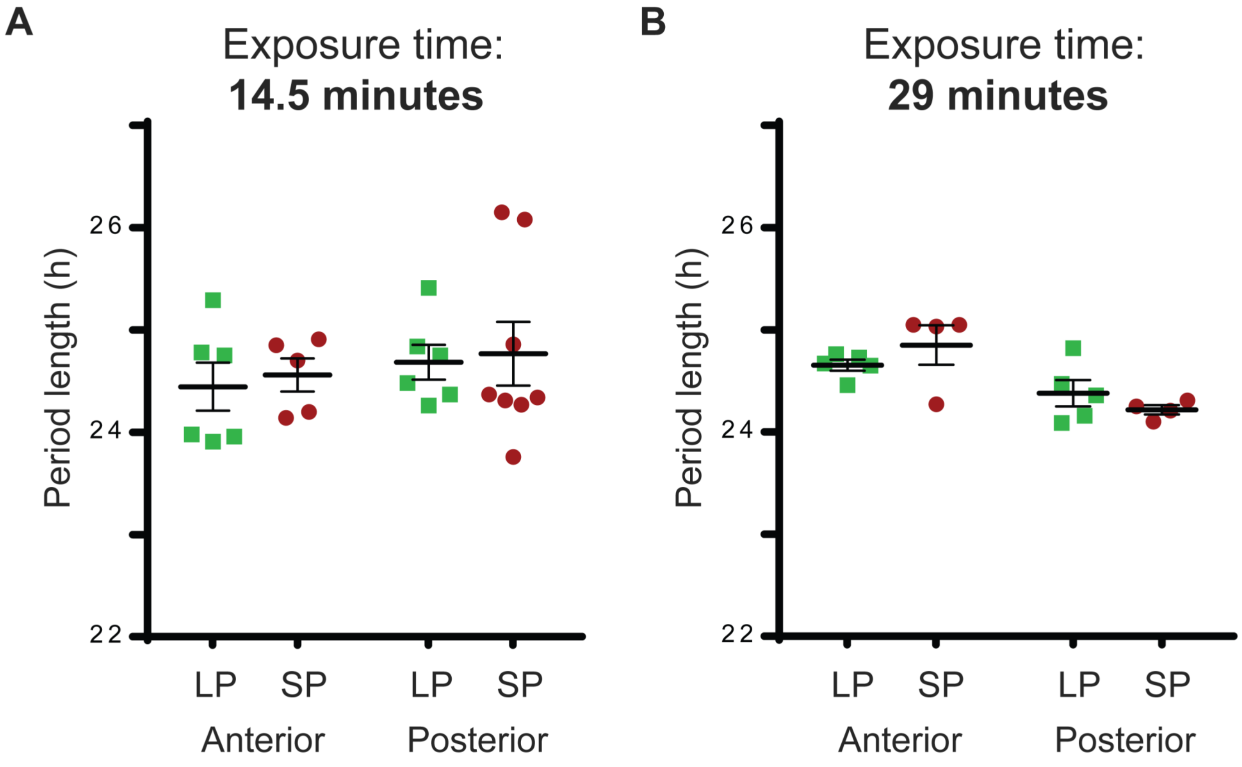

S4 Fig. Period of the PER2::LUC expression rhythms. Period was determined over three cycles and averaged per slice, for (A) the experiments with 14.5 minutes exposure time, (B) 29 minutes exposure time. Black bars indicate mean ± SEM.
