# Supplementary material for: Evidence for Weakened Intercellular Coupling in the Mammalian Circadian Clock under Long Photoperiod
Source: PLoS One. 2016 Dec 22;11(12):e0168954. doi: 10.1371/journal.pone.0168954 (PMC5179103; doi:10.1371/journal.pone.0168954)
Supplement: S5 Fig — Examples of PER2::LUC bioluminescence of six cells in LP (left) and SP (right) are shown, where the detrended signal of each individual cell (red) is compared to a sinusoid wave with the average period of the same cell (blue). The detrended signal is obtained by first smoothing the raw data. Then a fourth-order polynomial trend line was derived from the smoothed signal and subsequently subtracted. The sinusoidal signal was aligned to the half-maximum value of the rising edge of the first cycle. The time difference of the half-maximum values of the rising edge of subsequent cycles between the detrended data and the sinusoid represents the jitter in cycle-to-cycle interval. For each example, the number of the cell is shown in the upper right corner. Time is specified in ExT. (PDF) [file pone.0168954.s005.pdf]

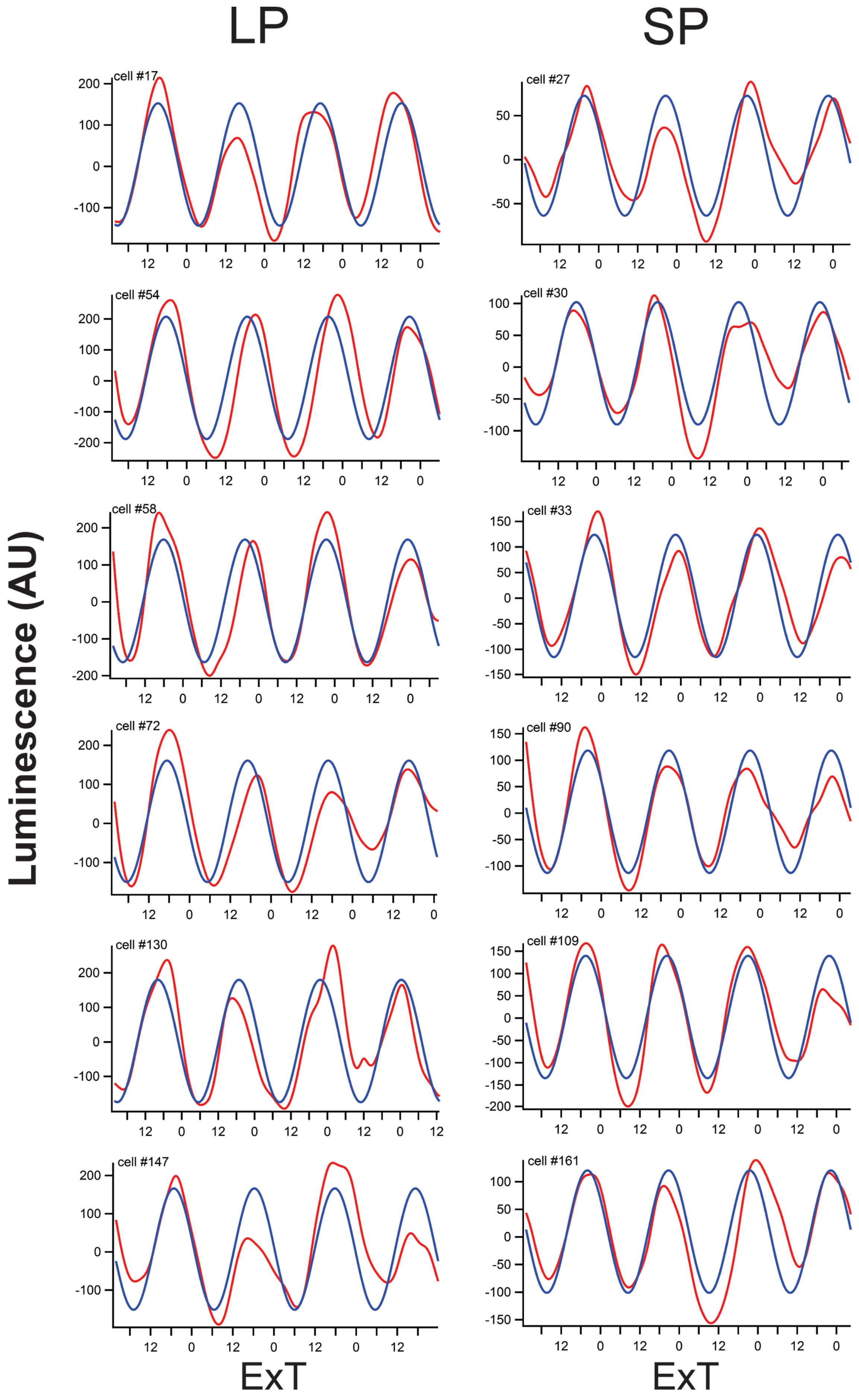

S5 Fig. Cycle-to-cycle jitter in cells is larger in long photoperiod. Examples of PER2::LUC bioluminescence of six cells in LP (left) and SP (right) are shown, where the detrended signal of each individual cell (red) is compared to a sinusoid wave with the average period of the same cell (blue). The detrended signal is obtained by first smoothing the raw data. Then a fourth-order polynomial trend line was derived from the smoothed signal and subsequently subtracted. The sinusoidal signal was aligned to the half-maximum value of the rising edge of the first cycle. The time difference of the half-maximum values of the rising edge of subsequent cycles between the detrended data and the sinusoid represents the jitter in cycle-to-cycle interval. For each example, the number of the cell is shown in the upper right corner. Time is specified in ExT.
